# Physical activity and temperature changes of Asian elephants (*Elephas maximus*) participating in eco-tourism activities and elephant polo

**DOI:** 10.1371/journal.pone.0300373

**Published:** 2024-05-02

**Authors:** Hannah B. Tilley, Derek Murphy, Kaja Wierucka, Tsz Ching Wong, Annaëlle Surreault-Châble, Hannah S. Mumby

**Affiliations:** 1 Applied Behavioural Ecology and Conservation Lab, Area of Ecology and Biodiversity, School of Biological Sciences, The University of Hong Kong, Hong Kong, Hong Kong SAR; 2 German Primate Centre—Leibniz Institute for Primate Research, Cognitive Ethology Laboratory, Göttingen, Germany; 3 Department for Primate Cognition, Johann-Friedrich-Blumenbach Institute, Georg-August-Universität Göttingen, Göttingen, Germany; 4 German Primate Center–Leibniz Institute for Primate Research, Behavioural Ecology and Sociobiology Unit, Göttingen, Germany; 5 Laboratoire Ethologie Cognition Développement, Université Paris Nanterre, Paris, France; 6 Le PAL, Saint-Pourçain-sur-Besbre, Allier, France; University of Mississippi, UNITED STATES

## Abstract

Captive and domestic animals are often required to engage in physical activity initiated or organised by humans, which may impact their body temperature, with consequences for their health and welfare. This is a particular concern for animals such as elephants that face thermoregulatory challenges because of their body size and physiology. Using infrared thermography, we measured changes in skin temperature associated with two types of physical activity in ten female Asian elephants (*Elephas maximus*) at an eco-tourism lodge in Nepal. Six elephants took part in an activity relatively unfamiliar to the elephants–a polo tournament—and four participated in more familiar ecotourism activities. We recorded skin temperatures for four body regions affected by the activities, as well as an average skin temperature. Temperature change was used as the response variable in the analysis and calculated as the difference in elephant temperature before and after activity. We found no significant differences in temperature change between the elephants in the polo-playing group and those from the non-polo playing group. However, for both groups, when comparing the average skin body temperature and several different body regions, we found significant differences in skin temperature change before and after activity. The ear pinna was the most impacted region and was significantly different to all other body regions. This result highlights the importance of this region in thermoregulation for elephants during physical activity. However, as we found no differences between the average body temperatures of the polo and non-polo playing groups, we suggest that thermoregulatory mechanisms can counteract the effects of both physical activities the elephants engaged in.

## 1. Introduction

Thermoregulation enables mammals to maintain their internal body temperature within a range that is vital for survival, independently of environmental temperatures [1] and during periods of physical exertion [2, 3]. Thermoregulation is particularly important during activity and exercise. During physical exertion, muscles require a burst of energy release allowing contraction and enabling swift movement; however, this also produces localised heat [4]. If exercise is intense or strenuous, an initial delay in heat loss regulatory mechanisms may result in an overall rise in core temperature [4]. If persistent, this has the potential to cause heat related illness, when the body is unable to dissipate the additional heat being produced and core temperature increases to levels which cause cell death or damage [5]. Exercise or exertion, which increases body temperature, can cause heat related illness or ‘hyperthermia’ in mammals [6, 7] including humans [4, 8].

To maintain a stable core body temperature during and following physical exertion, mammals use a wide variety of heat loss mechanisms [9] to prevent heat related illnesses. For example, some mammals, including humans [10] and equids [11] use evaporative cooling from sweating. However, most mammals are unable to sweat and rely on other mechanisms to thermoregulate, such as panting in canines [12] and some primates [13], or convection from the legs and other body parts (e.g., in Reindeer [14]). Other mammals which are unable to sweat, such as rabbits [15, 16], rats [17] and elephants, also have specialised highly vascularised extremities for heat dissipation from the skin [18].

‘Thermal windows’ are body regions, which aid with heat exchange and are characterized by an absence of hair [19, 20] and a dense network of blood vessels, with numerous connections between arteries and veins [21–25]. Examples of thermal windows include the facial sensory organs such as the nose, eyes and mouth [26, 27], appendages [28, 29], areas without hair such as the shoulder and between the shanks [26, 27] and the ears [16, 18, 30–33]. Blood flow to these regions is controlled through the vasoconstriction and dilation of blood vessels allowing heat loss to be regulated [20, 34, 35]. The predominant thermoregulatory challenge for these animals, especially in hot environments, is the dissipation of metabolic heat as it is produced through activity [36–39].

Heat dissipation is a particular challenge for elephants [18, 40–43] as in addition to the largest body mass of any terrestrial mammal and a low surface area to volume ratio, they also have the inability to regulate body temperature through sweating [42–44]. Elephants’ key physiological adaptation for heat loss is a heat storage mechanism whereby heat is accumulated during the day and released at night [40, 45]. Additionally, as with other mammals which utilise specific body regions for heat exchange, all three extant elephant species (*Elephas maximus*, *Loxodonta africana* and *Loxodonta cyclotis*) possess large ear pinnae which, through their large surface area to volume ratio and high vascularity, enable heat dissipation and act as thermal windows [27, 29, 46]. Elephants also engage in heat dissipation behaviours which include seeking shade, bathing in water, and flapping their ears continuously or intermittently to increase evaporative heat loss [20, 40, 47–50]. However, these behaviours can be limited in captive conditions [31, 51, 52].

Heat stress is a well-documented cause of death for wild Asian elephants [53, 54]. The demands of active metabolic heat dissipation and their size, coupled with the inability for elephants to engage in heat loss behaviours, also has the potential to cause hyperthermia in captive elephants, by exceeding their maximal survival limit leading to increased mortality [55]. Therefore, heat loss challenges should be viewed as a welfare concern in captive individuals. Using modelling techniques and two captive elephants, Rowe et al. [45] estimated that a potentially lethal core body temperature increase of ~8.0°C could occur in elephants after ~4 hours of continuous locomotion in the sun at >31°C. This research is particularly relevant for Asian elephants, where one in three individuals are captive (30–40% of the species) [56]. Many of these animals are used in tourism in hot tropical range countries; for physical activities such as elephant rides [57] or in sports such as elephant polo [58].

Elephant polo, like horse polo [59], has historically been a vigorous sport, with competitive players and elephants forced to run, turn rapidly and occasionally to physically engage with another elephant. The sport originated in Nepal in the 1980s and gained popularity in several South and Southeast Asian countries including Nepal, Thailand, India and Sri Lanka [58, 60]. The combination of activity and supporting the weight of two people (the elephant’s personal handler or ‘mahout’ and the polo player) has the potential to impact body temperature and cause heat related illness [4]. Furthermore, the use of equipment e.g., saddles, for activities such as polo could affect the temperature of certain distal regions more than others. Despite guidelines from the World Elephant Polo Association (WEPA) to promote elephant protection practices [60] and to address ongoing public welfare concerns, to our knowledge there is currently no literature examining elephant temperature regulation or physiological correlates of the physical exertion of this activity.

In this study, we used infrared thermography (IRT) to investigate the impact of the physical activity of polo on Asian elephant skin temperature. We compared the temperatures of a group of polo-playing elephants with a group which continued with other tourism activities (e.g., grass cutting and walks with tourists) which elephants were more familiar with. We investigated [1] whether different types of activity (polo or non-polo) differed in their impact on elephant skin temperature in different body regions and [2] whether there was variation across different body regions in skin temperature change after activity. We predicted that due to the physical exertion of the polo tournament (increased rate of muscular contraction from prolonged intense activity), elephants playing polo would show a greater increase in skin temperatures directly after exercise than elephants participating in other activities. We also predicted that the ear pinna would have a greater change in temperature than the other body regions sampled, as it is a recognised thermal window and utilised for heat dissipation in elephants.

## 2. Materials and methods

### 2.1 Elephants & activities

Our study was conducted with ten female Asian elephants (*Elephas maximus*; aged between 15–60 years). The animals were housed at Tiger Tops Tharu Lodge (27°34’10.5"N 84°06’06.9"E), an eco-tourism lodge on the border of Chitwan National Park in Nepal. This area is within the natural range of this species, with 25–30 wild individuals resident within the Chitwan region [61, 62]. Elephants were housed singly (n = 4) or with one other individual (n = 6) in fenced outdoor enclosures and cared for by a team of registered veterinarians and their mahouts (‘carers’; two per elephant) who were responsible for their daily care, tourism schedules [63] and who rode the elephants during activities. As part of their regular tourism schedule from September-May, elephants participated in eco-tourism activities including grass cutting (walking to the site, carrying cut grass) and forest walks (see S8.1 Appendix in S1 File). Additionally, elephants infrequently compete (every 2–3 years, with the previous tournament in 2016) in a week-long elephant polo competition. This is only undertaken within the winter period (in December) to avoid the adverse weather and high temperatures of monsoon season (May-September).

#### 2.1.1 Elephant polo

The lodge chose six elephants to participate in the six-day polo competition (1^st^-6^th^ December 2019), while four continued with other activities (grass cutting and tourism activities) during the competition period. All polo playing elephants had participated in the activity previously.

The elephant polo tournament consisted of six matches, which were played on a grass pitch with three elephants on two teams [60]. During the game the elephants were ridden by two people: their mahout and a polo player. The mahout sat astride the elephant’s shoulders (axilla) and provided verbal and physical commands/signals with their feet against the elephants’ neck to direct the animal throughout the course of the game [58, 64]. Players sat on a saddle (placed just behind the elephants’ axilla and proceeding down the back preceding the elephants’ hip bones) and used a long wooden mallet to direct the ball into the opposition’s goal [64]. Each match within the tournament was divided into two 10-minute halves with a 15-minute interval for player and elephant recuperation [60].

#### 2.1.2 Other tourism activities

Familiar activities (grass cutting/ trekking) were on average 2.5 hours in duration during which elephants walked at a leisurely pace with frequent pauses to enable tourists to take photos and enjoy the experience. Elephants undertook grass-cutting in the morning and trekking in the afternoon. Both activity types involved periods where elephants were in sunlit and shady conditions.

### 2.2 Data collection

We used IRT for measuring the skin temperature of elephants from different body regions. This non-invasive [65–67] and immediate tool [68] provides a representation of the surface temperature distribution of the body by recording the naturally emitted infrared radiation from the surface of the skin [69–71]. We took lateral thermograms (to prevent errors due to angle distortion) during the polo tournament in December 2019 using a FLIR One Pro iPhone camera (FLIR Systems, Inc., Wilsonville, OR, USA) with a spectral range of 8–14 μm and a thermal sensitivity of 150 mK and an accuracy of ±3°C. To account for the effects of direct sunlight on temperature measurements [see 72] we captured thermograms at a 3.5–4 m distance, while elephants were standing within or immediately outside of enclosures in the shade. We took thermograms of all elephants (including non-polo playing elephants) prior to (~07:15) and after polo matches on each of the polo tournament days (either ~13:00 or ~17:30). For five days of the tournament elephants played only in matches for a half day (finishing at ~13:00) however on the second day of the tournament elephants played for a full day (finishing at ~17:30). We captured all elephant thermograms when individuals were not being handled or restrained by mahouts and within 10-minutes of the end of the activity, to enable the researcher in charge to move between elephant enclosures. Each time thermograms were taken, several pictures were initially recorded of each elephant and one selected for the study. The thermograms selected showed elephants in a static pose with no parts of the animal moving and with the ears lying flat against the body. We obtained local ambient temperature data from online weather reports for the region [73].

**Fig 1:**
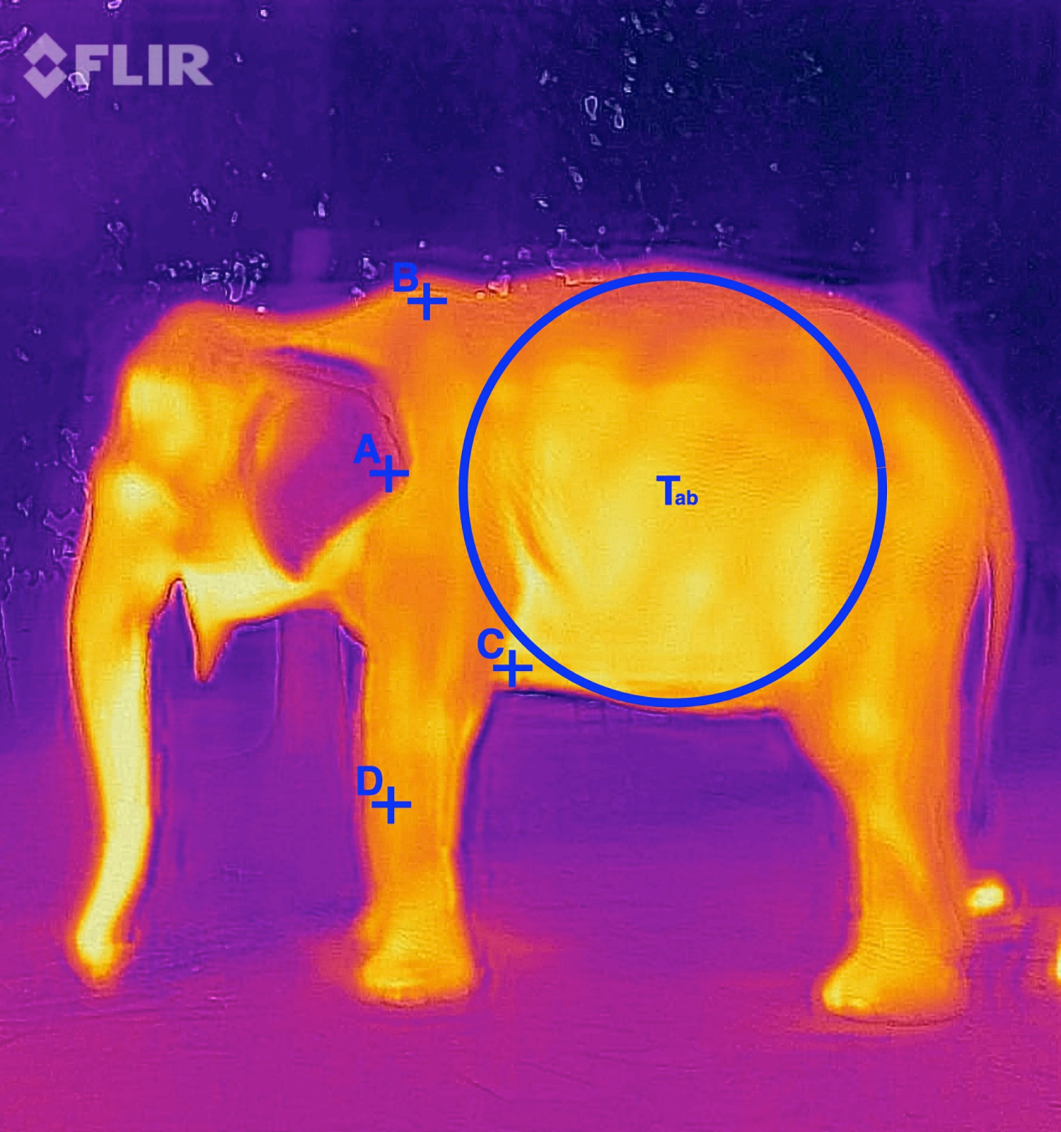
Lateral thermogram depicting regions where skin temperature data were collected for each elephant. Body regions sampled included (A) the pinna of the ear, the shoulder (B), axilla (C), and foreleg (D) in addition to the average body temperature (T_ab_) for each subject.

We extracted temperature data using FLIR Tools (FLIR Systems, Inc., Wilsonville, OR, USA). The software allows for taking readings of specific pixels (points) or an average skin temperature of a greater area. We extracted thermal data for four body regions: the outer point of the ear pinna (“pinna”), the top of the shoulder (“shoulder”), the axilla and the midpoint of the radius on the outer leg on the side where the photo was taken (“foreleg”) (Fig 1). Markers were positioned 4–5 pixels from the outline of the elephants’ body into the measured body region to avoid edge effects and promote consistency across images. We selected the pinna as it has been identified as a thermal window in elephants [33]. We chose the shoulder and the axilla as these areas are affected by riding or riding equipment, such as saddles [74, 75], which could impact the temperature of these areas from resulting friction, insulation or abrasion. In horses, differential tissue heating from saddle wear has been found to cause muscle pain and atrophy [76]. We selected the foreleg to account for the increased exertion and weight-bearing during exercise. We chose the foreleg over the hindleg as greater temperature changes have been found in horse forelegs compared to their hindlegs [77] and Asian elephant limb motion is more similar to horses than to other mammals [78]. We did not include points for the head or the proboscis as independent areas for heat exchange could not be differentiated in these areas with African Savannah elephants [33]. We obtained the average body temperature measurement of the skin (henceforth: average body temperature) using the area measurement tool in FLIR Tools, which gives the mean temperature within a selected area in an image. In this study, we selected a circular area between the elephant’s lower abdomen, top of the thoracic vertebrae, tricep muscle and tensor fasciae muscle (Fig 1).

### 2.3 Data analysis

We conducted a preliminary linear mixed effected model (LMM) with temperature after activity as the response variable and day length (half or full day) and body part as explanatory variables S2 Table in S1 File, supplementary information). However, this showed no effect of day length. Therefore, we pooled the data for both the half and full days.

We used a LMM to examine differences in skin temperature between polo and non-polo playing elephants before and after physical activity (*lme4* package [79]). Temperature change (response variable) was calculated as the difference between skin temperature before and after activity for each body region separately (for polo playing and non-polo playing elephants). We included polo participation (binary) and body region (factor with 5 levels; average body temperature, axilla, shoulder, foreleg and pinna) as fixed effects with an interaction between these two variables. Elephant identity and date were included as random intercepts. Statistical analyses were performed using R, version 4.0.5 [80].

Initially we ran the model with an interaction between the type of activity (polo; yes or no) and body part as fixed effects, however after performing a likelihood ratio test, we found no evidence that temperature change within body parts differed between polo-playing and non polo-playing elephants (F(1,4) = 0.61, p = 0.65). Therefore, to examine these effects separately we removed the interaction term and reran the model, with body part and polo practice as fixed effects.

### 2.4 Ethical considerations

This research was approved by the Committee on the Use of Live Animals in Teaching and Research (CULATR 5192–19) at The University of Hong Kong on 5^th^ November 2019.

This study was conducted during an elephant polo tournament already taking place at the study site, offering an opportunity to collect non-invasive thermal data during a time of consistent physical activity. The tournament was not undertaken at the request of the authors; however, Tiger Tops Tharu Lodge gave full permission for research to be carried out alongside the tournament. The organisers implemented strict welfare and animal ethics protocols throughout, which prohibited the use of sticks or hooks to motivate elephants and ensured that elephants had a rest period and were provided food and water. The organisers contacted the authors to examine elephant welfare regarding the polo tournament.

## 3. Results

We took a total of 98 images for 10 elephants (μ = 10; min = 6; max = 12, per elephant). Due to radiometric data loss in some images, we used 89 for analyses (58 for polo-playing elephants and 31 for elephants which were not chosen to play in the polo tournament). The minimum temperature we recorded was for the ear pinnae (3.3°C) of a non-polo elephant and the maximum temperature we recorded was for the foreleg (37.9°C) of the same elephant (μ = 23.3°C for all photos) (see S1 Table S8.2 Appendix in S1 File for average temperatures for each body part before and after activity). Average ambient temperature before activity was recorded at 15°C (min = 14°C; max = 16°C) and after activity at 23.8°C (min = 21°C; max = 26°C).

### 3.1 Effects of activity on elephant body temperature

Our model results showed no effect of type of activity (elephant polo or normal tourism activities) on temperature change of elephants. The estimated standard deviations for the contributions of the random effects were, for individual ID, SD = 1.67, and for date, SD = 1.94.

### 3.2 Temperature differences between body regions of elephants undergoing activity

**Fig 2:**
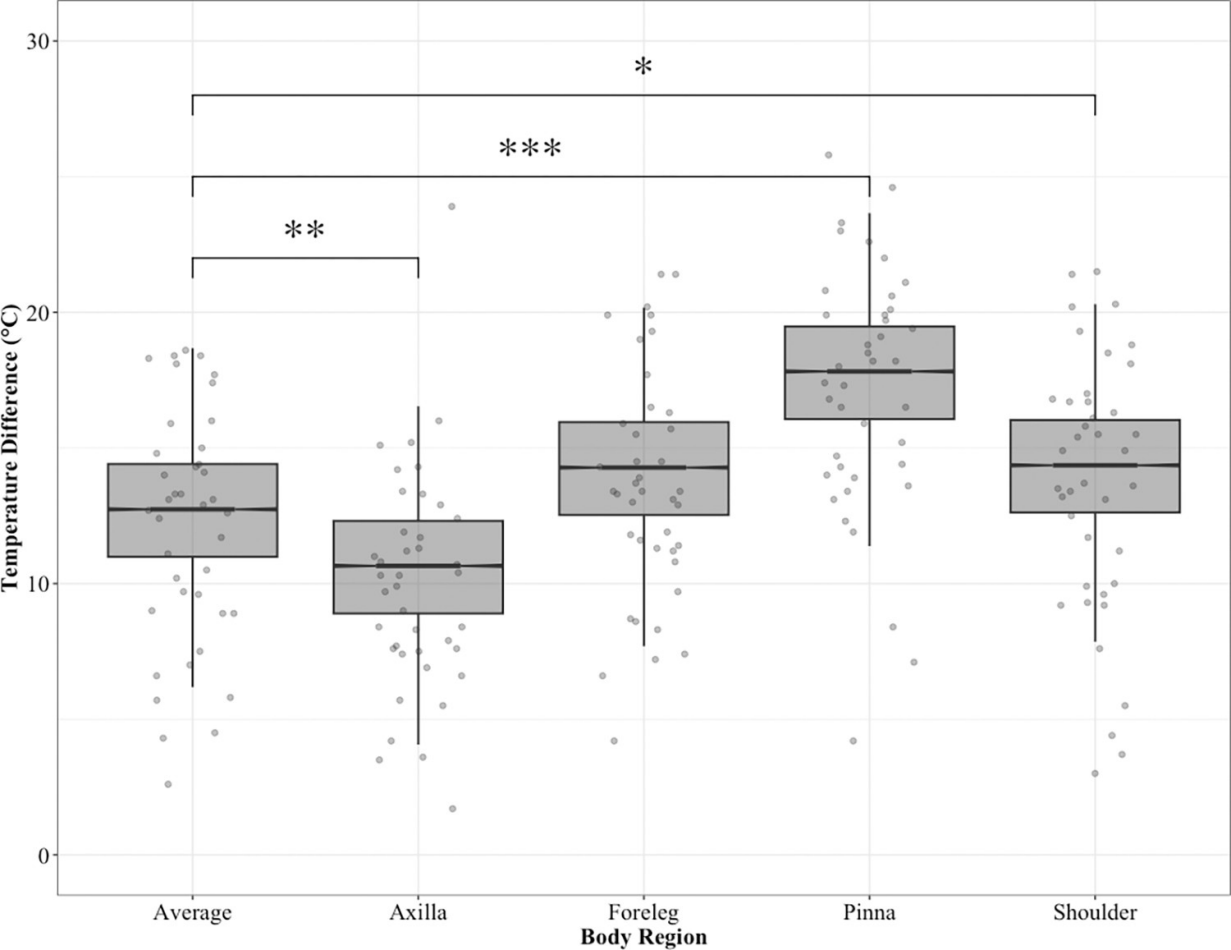
Model predicted skin temperature change for elephants undergoing activity (both polo and other tourism activities) (n = 89 for each body part). Midline indicates median and boxes show 1^st^ and 3^rd^ quartiles. Whiskers indicate the 95% confidence interval. (Significance levels indicated *p<0.05, **p<0.01, ***p<0.001).

For all elephants undergoing activity (polo and non-polo playing elephants), we found significant differences in skin temperature change (before and after activity) between the average body temperature and several of the other body regions (Fig 2). Our model results show significant differences in skin temperature change between average body temperature and the axilla (t(181) = -2.63, β = -2.1, *p* = 0.009), the ear pinna (t(181) = 6.25, β = 5, *p* = <0.0001) and the shoulder (t(181) = 2, β = 1.6, *p* = 0.04). However, we found no significant difference between the average body temperature and the foreleg (S3 Table in S1 File, supplementary information).

## 4. Discussion

In this study, we investigated how physical activity impacted skin temperature of captive Asian elephants. We collected thermal data from a group of elephants that were used in an activity they rarely engaged in–an elephant polo tournament–and from another group that did not play polo but continued with their usual eco-tourism activities. We found that skin temperatures in body regions increased in both groups after activity, with no significant differences associated with the two different activities. This suggests that either elephant physiological adaptations were sufficient to cope with these activities or that one activity was not more strenuous than the other. When comparing the change in average body temperature with that of the other body regions, our results showed that the greatest change in skin temperature (before and after activity) was found in the ear’s pinnae but that there were also significant differences in the axilla (smaller change in temperature) and in the shoulder (greater change in temperature). Unexpectedly, we found no difference between the skin of the average body temperature and the foreleg of polo playing and non-polo playing elephants.

Our findings indicate that the polo tournament did not have a greater impact on elephant skin temperature than usual eco-tourism activities. This may mean that the different types of exercise were comparably strenuous or that elephants are well-adapted homeotherms and able to cope with the temperature increase from the activities (over the short length of time which elephants were engaging in it). Elephants have the capacity to deal with extreme heat and can maintain homeothermy in range environments with high ambient temperatures [81]. In the wild, Asian elephants regularly engage in intense periods of activity, with home range sizes from ~50 km^2^ to >250 km^2^ and some populations undertaking additional seasonal migrations of ~140 km [82–88]. It is possible that both activities that our population experienced did not pose any greater heat stress than normal activity levels of this species and that physiological thermoregulatory adaptions enabled elephants to cope with both activity types.

Force was not used to motivate elephants playing polo, which may explain the lack of difference between polo playing elephants and those engaged in other activities. In line with elephant welfare concerns voiced by animal activist groups, tour operators and the public [89–91], the field site prohibits several common practices in SE Asia concerning motivating elephants. Mahouts are prohibited from carrying the ankus, also known as a bullhook or guide which is often used to motivate elephants to increase their speed or obey certain commands [92]. The absence of this item to force elephants to comply to run, perform tight turns or physically engage with another animal, incidents often documented in horse polo [59], was not witnessed by authors during the elephant polo tournament. Elephants walked at a brisk pace throughout but were not forced to into excessive physical exertion, which was in line with polo rules imposed by site owners (DB Chaudhary, pers. comm). Therefore, it is possible that activities were equally strenuous which is why no significant temperature difference was observed between polo and regular tourism activities.

Of the body regions sampled, we found that the ear pinna had the greatest skin temperature change compared with that of the body. These results are in keeping with thermoregulatory research on elephants, which has found that elephants rely on non-evaporative strategies including specific organs to transmit heat [18, 27, 30]. Despite early research disputing the ear as a heat loss mechanism [93], research has emphasised the importance of ears in thermoregulation and heat dissipation in other mammalian species [16, 32]. This includes African elephant research [18, 30, 31, 33], where 100% of the radiative heat loss was found to be from the ear pinnae [27], and Asian elephant research [33, 94–96]. It unsurprising that the skin temperature change of the pinnae is greater than each of the other body regions, as we expect that elephants would utilise this thermal window to dissipate heat more than other regions that are less specialised for heat loss. Furthermore, the skin temperature changes of the elephants’ pinnae are not consistent with results for the average core body temperature (T_ab_). This suggests that specialised thermal windows, such as the elephants’ ears [33, 94, 95], were successfully used to dissipate heat to maintain body temperature within an optimal range.

Differences were also recorded between the change in average body skin temperature and that of other body regions. We recorded that the shoulder had a greater temperature difference than the skin average body temperature. This was expected as this body part directly engages with riding and equipment used in eco-tourism activities. For both polo and non-polo activities, the mahout sits on the shoulders of the elephants [58, 64], giving him control over the elephant’s movements. The added body temperature and weight of the mahout may have affected the temperature of this region. In studies with racehorses, the presence of a rider increases the temperature of several different body regions (neck, front and middle) simultaneously [97]. Additionally, riding and the equipment facilitating equine leisure activities such as the girth [98] and the saddle [99, 100] impact the thermography of horses. Conversely, the axilla had a smaller temperature change than the average body temperature. As the axilla is located closer to the body’s core it is possible that this region displays narrower temperature variations than other regions of the body. Although this has yet to be studied in gigantotherms, large proximal-distal temperature gradients have been recorded in other active mammals [101]. Limbs are peripheral body regions (regional heterothermy) with more variable temperatures to that of the core body [101, 102]. However, due to their locomotory function and high muscular density, limbs produce more heat during activity and therefore operate at the same or at a slightly higher temperature than of the core body during exercise [101, 102]. This may explain the lack of temperature change differences between the skin of the foreleg and that of the average body temperature found here and the similar before and after temperatures of these regions.

Our results show no difference in body temperature changes between the two activity types, suggesting that if body temperature is to be considered a marker for welfare, we found no evidence that polo playing has any more impact on this marker than the other eco-tourism activities which elephants engage in. We recorded elephant skin temperatures of above 30°C after regular activities and elephant polo, the temperature at which elephants adjust their behaviour (e.g. seeking shade and water) to reduce environmental heat load [81]. Although these recorded temperatures have the potential to be harmful to elephants, the short periods of play for polo and rest times for both activities mean we do not see issues in terms of thermoregulation alone concerning the elephants engaging in these forms of exercise. However, to explore the welfare implications of polo more thoroughly, other behavioural and physiological indicators, such as serum cortisol or faecal glucocorticoid metabolites should be investigated to provide a more comprehensive study of the welfare implications of polo.

Our study has some methodological limitations and technical constraints. We used a single point on one thermogram pixel to assess different body regions. Further work (e.g., following the methodology 94) should gather points over a larger area of each body region, such as the ears. For example, elephants have multiple thermal windows in each ear [33, 94], and can manipulate vasoconstriction or vasodilation in each ear separately [33].

## 5. Conclusion

We used infrared thermography to compare skin temperature changes in Asian elephants engaging in an elephant polo tournament with those undertaking familiar eco-tourism activities. We found no significant differences in skin temperature change between polo-playing and non-polo-playing elephants. However, this could be associated with the specific practices and rules to limit the impact of polo on these animals at this specific site and event. Although temperature differences were observed between several of the distal regions, elephant ear pinnae showed the greatest changes in temperature from before to after activity irrespective of whether elephants had been in the polo competition. This result supports previous findings and indicates the importance of the ears’ role in elephant thermoregulation.

## Supporting information

S1 File (DOCX)

S1 Appendix Datasheet for elephant thermograms for different activity types (“Master datasheet 09092022.csv”). (XLSX)

S2 Appendix Full and Half day analystical comparison of elephants undergoing activity (“Full_Half.csv”). (XLSX)

S3 Appendix R Script of elephant thermogram analysis (“Therography Script_Submission”). (R)

S4 Appendix Data dictionary with variable explanation (“Data Dictionary”). (TXT)
