# Supplementary material for: Physical activity and temperature changes of Asian elephants (Elephas maximus) participating in eco-tourism activities and elephant polo
Source: PLoS One. 2024 May 2;19(5):e0300373. doi: 10.1371/journal.pone.0300373 (PMC11065253; doi:10.1371/journal.pone.0300373)
Supplement: S1 File — (DOCX) [file pone.0300373.s001.docx]

**S8.1 Appendix**

**Methods- Elephant Information**

Elephants in the study were familiar with close human interaction such as being touched and obeying commands from their mahouts. Elephants undertook several different types of activities with tourists including supervised feeding, tours to areas where grass was cut to feed elephants and walks into or around the periphery of the National Park. Grass cutting was usually a longer activity for up to five hours whereas the other walking activities were at most 2 hours in length. During the grass cutting, elephants would hike to the grass cutting site with tourists following behind. Once at the site tourists would learn to cut the grass and elephants would then carry it back to Tiger Tops for later consumption. Due to the high welfare standards of the site, elephants were not chained at any point and were instead kept in large enclosures called ‘corrals’ either singly (n=4) or in pairs (n=6). Additionally, tourists were not permitted to touch or ride elephants, although on walks tourists were allowed to walk next to elephants.

Mahouts typically fed elephants twice per day (~6:00 and ~13:00) on grasses taken from the surrounding areas of grassland and a staple food called ‘kuchi.’ Kuchi parcels consist of grasses, molasses, chickpeas, rock salt and rice paddy and 50 were given to the elephants in each feeding session. Elephants were also allowed to naturally forage as they engaged in tourism activities. Their diet was additionally substituted with seasonal foods such as sugar cane and banana trees and various fruits including apples and bananas.

**S8.2 Appendix**

**Results**

**Table S1:** **mean skin temperatures for each body part investigated before and after the two different activity types.**

|  | **Polo Tournament** | | **Other Activities** | |
| --- | --- | --- | --- | --- |
| **Body Part** | **Before** | **After** | **Before** | **After** |
| **Average** | 18.2 | 29.6 | 18.1 | 32.0 |
| **Axilla** | 20.2 | 30.9 | 20.7 | 31.5 |
| **Foreleg** | 16.4 | 29.4 | 16.5 | 31.7 |
| **Pinna** | 11.6 | 28.1 | 11.3 | 29.2 |
| **Shoulder** | 15.5 | 28.6 | 16.1 | 30.9 |

**Table S2:** **Coefficients and significance values for the LMM model investigating differences in elephant temperature after activity between full and half activity days.** Statistical significance (*p* < 0.05) is indicated in bold.

| Model | Estimate | | Standard Error *(SE)* | | *t-*value | | *p-*value | |
| --- | --- | --- | --- | --- | --- | --- | --- | --- |
| (Intercept) | | 29.635 | 0.772 | 38.377 | | **<0.0001** | |  |
| Full/Half | | 0.965 | 0.528 | 1.827 | | 0.069 | |  |
| Body region- Axilla | | 0.737 | 0.712 | 1.035 | | 0.302 | |  |
| Body region- Foreleg | | -0.172 | 0.712 | -0.242 | | 0.809 | |  |
| Body region- Ear Pinnae | | -2.062 | 0.712 | -2.894 | | **<0.004** | |  |
| Body region- Shoulder | | -1.057 | 0.712 | -1.484 | | 0.139 | |  |

**Table S3:** **Coefficients and significance values for the LMM model investigating average temperature change between elephants from the polo group and the non-polo group.** Statistical significance (*p* < 0.05) is indicated in bold.

| Model | Estimate | | Standard Error *(SE)* | | *t-*value | | *p-*value | |
| --- | --- | --- | --- | --- | --- | --- | --- | --- |
| (Intercept) | | 14.021 | 1.416 | 10.121 | | **<0.0001** | |  |
| Polo Group | | -2.277 | 1.238 | -1.851 | | 0.098 | |  |
| Body region- Axilla | | -2.125 | 0.807 | -2.632 | | **0.009** | |  |
| Body region- Foreleg | | 1.510 | 0.807 | 1.870 | | 0.063 | |  |
| Body region- Ear Pinnae | | 5.052 | 0.807 | 6.258 | | **<0.0001** | |  |
| Body region- Shoulder | | 1.615 | 0.807 | 2.000 | | **0.046** | |  |
